# Supplementary figures and images for: Elastin-targeted nanoparticles delivering doxycycline mitigate cytokine storm and reduce immune cell infiltration in LPS-mediated lung inflammation
Source: PLoS One. 2023 Jun 2;18(6):e0286211. doi: 10.1371/journal.pone.0286211 (PMC10237374; doi:10.1371/journal.pone.0286211)

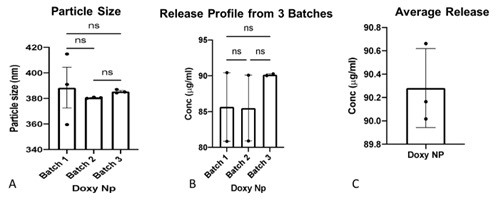

Supplement: S1 Fig — (JPG) [file pone.0286211.s001.jpg]

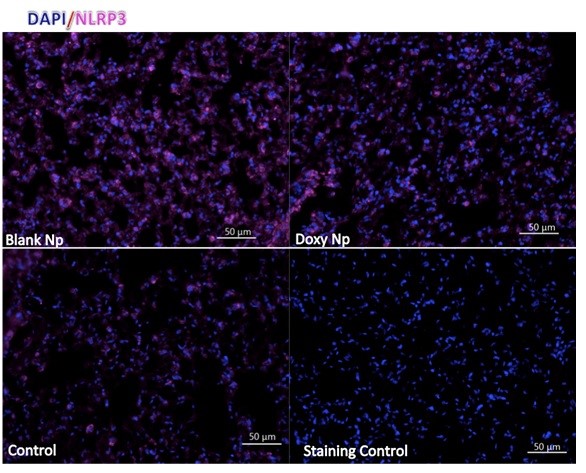

Supplement: S2 Fig — (JPG) [file pone.0286211.s002.jpg]

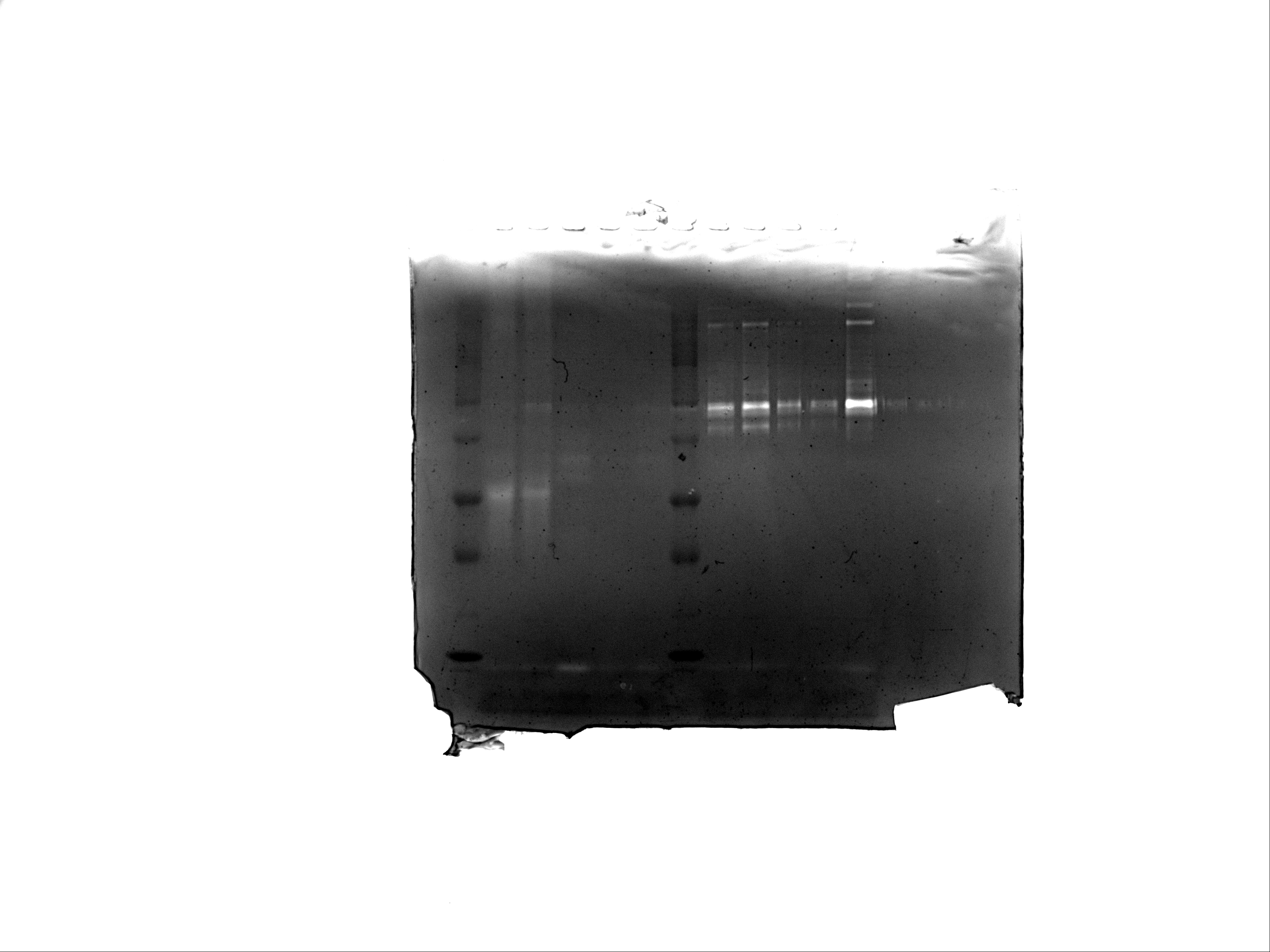

Supplement: S1 Raw images — (ZIP) [file pone.0286211.s003.zip › gel1 300ms.TIF]

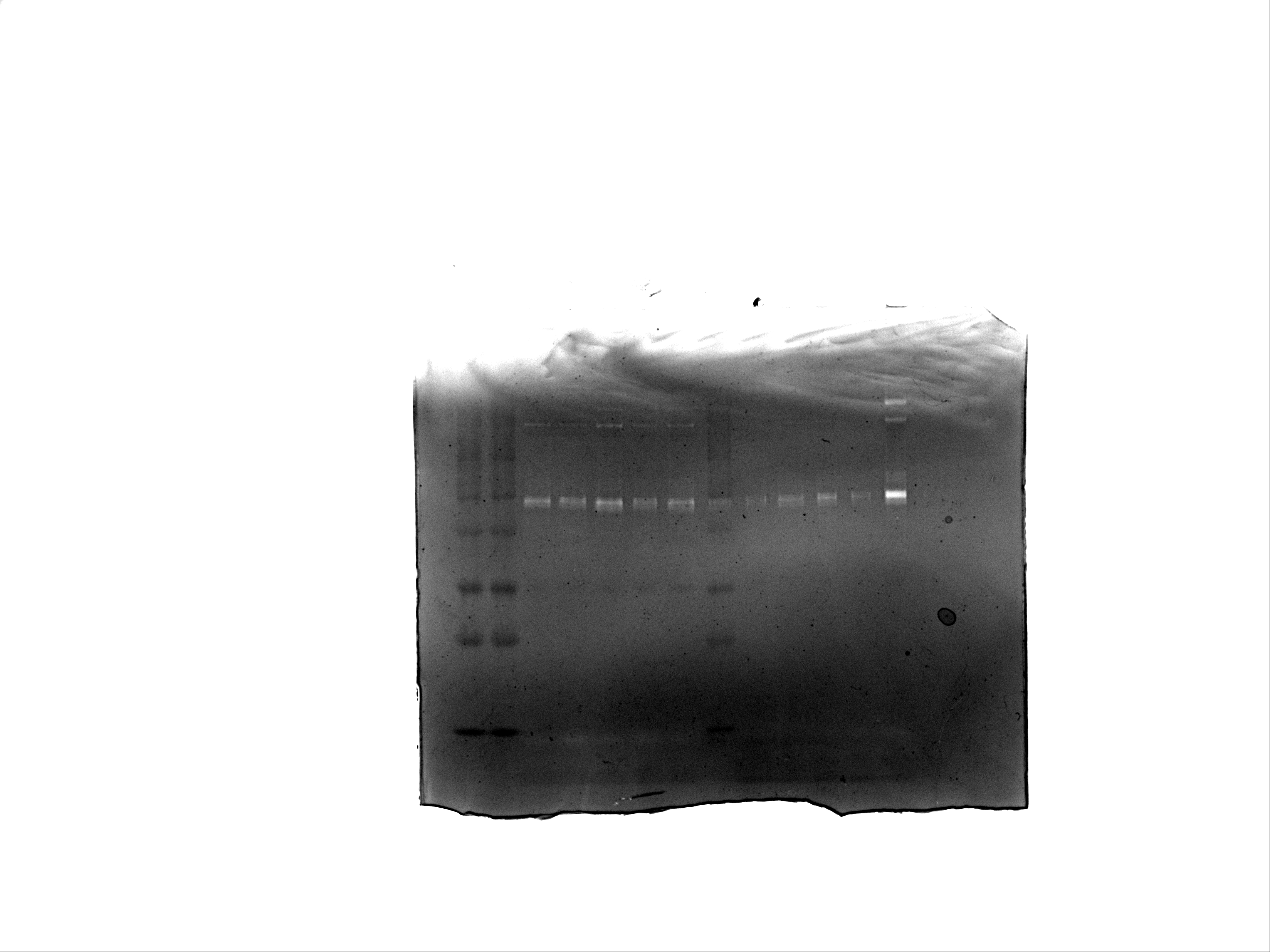

Supplement: S1 Raw images — (ZIP) [file pone.0286211.s003.zip › gel2 300ms.TIF]
