# Supplementary material for: Elastin-targeted nanoparticles delivering doxycycline mitigate cytokine storm and reduce immune cell infiltration in LPS-mediated lung inflammation
Source: PLoS One. 2023 Jun 2;18(6):e0286211. doi: 10.1371/journal.pone.0286211 (PMC10237374; doi:10.1371/journal.pone.0286211)
Supplement: Attachment — Submitted filename: Response to Reviewers revised manuscript.docx [file pone.0286211.s005.docx]

We again thank the reviewers for their comments and suggestions.

**Reviewer #1**: The authors have addressed most of the points. I have two more minor comments -
*1. Kindly include the justification to comment number 1 (Reviewer 1) in the introduction section to highlight more on the scope of the current study.*

Response: As per Reviewer’ suggestion we have included the justification to comment number 1 (Reviewer 1) in the introduction section to highlight more on the scope of the current study on Page Number 4, in paragraphs 1 and 2.

*2. Kindly rearrange the x- and y- axis of the Figure 4Aiii and 4Aiv. Whatever, justification the authors given to this point, they did not follow this on Figure Av, Avi and Avii. The entire population acquired should be visible. There is no question about the analysis. But the representation is not adequate. The authors can easily fix this with the help of T button in the FlowJo.*

Response: As per reviewer’ suggestion we have made changes in the Figure4Aiii and 4Aiv and have rearranged the x- and y- axis of the Figure 4Aiii and 4Aiv to include the entire population.
